# Supplementary material for: Mining Sources of Resistance to Durum Leaf Rust among Tetraploid Wheat Accessions from CIMMYT’s Germplasm Bank
Source: Plants (Basel). 2022 Dec 22;12(1):49. doi: 10.3390/plants12010049 (PMC9823849; doi:10.3390/plants12010049)
Supplement: Supplementary file 1 [file plants-12-00049-s001.zip › plants-2031394-supplementary.pdf]

Table S1. Intrid and cross or cultivars and seed source origin of durum cultivars tested at seedling in the greenhouse and field of selected seedling resistant.

Tetraploid accessions		
		Country
Intrid[a]	Cross or cultivar	Origin (seed)
G5375	RL6010/6*YR//2*STIL/MEX75	CIMMYT
G5369	RL6010/6*YR//2*STIL/MEX75	CIMMYT
CWI20891	RECIO DE MALAGA	SPAIN
CWI22027	FAO29.934	CYPRUS
DW5345	KKC/1381.2	TURKEY
G5359	DWL5023/CNO79	CIMMYT
G5392	RL6043/3*NAC//2*TCHO	CIMMYT
DW15671	SOMAT_3//SOOTY_9/RASCON_37/3/BINTEPE 85/SULA	CIMMYT
G5364	DWL5023/CNO79	CIMMYT
DW15675	AINZEN_1/6/2*CMH82A.1062/3/GGOVZ394//SBA81/PLC/4/AAZ_1/CREX/5/HUI//CIT71/CII	CIMMYT
CWI21151	AZIZIAH 12565	PORTUGAL
CWI20528	HARLAN J.R 5719	TURKEY
CWI23230	PARKER JH 52	ETHIOPIA
CWI22327	HARLAN J.R 14	JORDAN
CWI32536	ATSIKI 3	GREECE
G6857	CMH83.2900	CIMMYT
G3543	H567.71/CIT71//2*MEX75/2*SHWA	CIMMYT
G8743	SN64/3*CIT71	CIMMYT
CWI22214	IAR.W.92.1	ETHIOPIA
CWI23460	ELS6404.156.2	ETHIOPIA
DW7085	BROWN SEEDED A1-124	CIMMYT
G8731	CMH82A.1056	CIMMYT
G5235	TDIC1458/MEX75//CIT71/T.BOEOTICUM	CIMMYT
G5207	T.DIC 1460/MEX75//MEXI75/T.MON.2433	CIMMYT
G5394	RL6043/3*NAC//2*TCHO	CIMMYT
DW15673	SOMAT_3.1//WODUCK/CHAM_3/5/AJAIA_16//HORA/JRO/3/GAN/4/ZAR	CIMMYT
G8505	DWL5023/NAC	CIMMYT
CWI22423	ISWRN 68-443/GA-A7, PI434878	SOUTH AFRICA
CWI21666	G46049, PI264438	GREECE
CWI20476	HARLAN J.R 4460	TURKEY
CWI22683	CItr 3754	RUSSIA
CWI22087	IAR.W.11.2	ETHIOPIA
CWI21791	CANDICANS 76.10	RUSSIA
CWI22053	DUBBIE	ISRAEL

G5208	T.DIC 1460/MEX75//MEX75/T.MON.2433	CIMMYT
G5424	CIT71/2*T.CARTHLICUM	CIMMYT
DW649	CRA/P66.270	CIMMYT
G3988	T.DIC 1460/MEXI75	CIMMYT
CWI355	CALIFORNIA ALCALA REAL	MEXICO
G5421	CMH77A.682/YAV79	CIMMYT
CWI23606	QUALSET BYDV 6	USA
CWI21696	PI265009	YUGOSLAVIA
G8813	KHP*4/D31708	CIMMYT
DW7103	WHITE SEEDED K2-152	CIMMYT
CWI22175	IAR.W.63.1	ETHIOPIA
CWI20114	BEYAZ KAPCIK	TURKEY
CWI23067	ABYSSINIAN 31	ETHIOPIA
CWI20425	HARLAN J.R 4497	TURKEY
CWI21256	FELLOWS P8741	ETHIOPIA
CWI22139	IAR.W.42.3	ETHIOPIA
CWI22166	IAR.W.56.2	ETHIOPIA
CWI22201	IAR.W.84.2	ETHIOPIA
CWI22089	IAR.W.12.2	ETHIOPIA
CWI22102	IAR.W.22.3	ETHIOPIA
CWI22280	IAR.W.174.3	ETHIOPIA
CWI22064	IAR.W.1.1	ETHIOPIA
CWI22294	IAR.W.185.1	ETHIOPIA
CWI22250	IAR.W.131.1	ETHIOPIA
CWI23359	MORISCO	SPAIN
DW3139	GORDEIFORME SARI BUGDAY	TURKEY
CWI23446	ELS6404.139.7	ETHIOPIA
G6904	CMH84.1106	CIMMYT
CWI21737	HARLAN J.R 1939	ETHIOPIA
CWI23440	ELS6404.131.3	ETHIOPIA
CWI22143	IAR.W.44.2	ETHIOPIA
CWI23065	ABYSSINIAN 26	ETHIOPIA
CWI23385	ELS6404.84.3	ETHIOPIA
CWI23473	ELS6404.61.2	ETHIOPIA

Intrid[a] = CIMMYT Germplasm Bank accession number

Table S2. Intrid and cross or cultivar and seed source origin of tetraploid accessions tested against 15 leaf rust races at seedling stage in the greenhouse

Intrid	Cross or cultivar	Country Origin (seed)
DW7085[a]	BROWN SEEDED A1-124	CIMMYT
DW7103	WHITE SEEDED K2-152	CIMMYT
DW15671	SOMAT_3//SOOTY_9/RASCON_37/3/BINTEPE 85/SULA	CIMMYT
DW15673	SOMAT_3.1//WODUCK/CHAM_3/5/AJAIA_16//HORA/JRO/3/GAN/4/ZAR	CIMMYT
DW15675	AINZEN_1/6/2*CMH82A.1062/3/GGOVZ394//SBA81/PLC/4/AAZ_1/CREX/5/HUI//CIT71/CII	CIMMYT
G3988[b]	T.DIC 1460/MEX75	CIMMYT
G5424	CIT71/2*T. CARTHLICUM	CIMMYT
G8731	CMH82A.1056	CIMMYT
G3543	H567.71/CIT71//2*MEX75/2*SHWA	CIMMYT
G6857	CMH83.2900	CIMMYT
G6904	CMH84.1106	CIMMYT
DW7147	DULAX 2	CIMMYT
G5359	DWL5023/CNO79	CIMMYT
G5364	DWL5023/CNO79	CIMMYT
G5207	T.DIC 1460/MEX75//MEX75/T.MONOCOCCUM.2433.	CIMMYT
G5369	RL6010/6*YR//2*STIL/MEX75	CIMMYT
G5375	RL6010/6*YR//2*STIL/MEX75	CIMMYT
G5394	RL6043/3*NAC//2*TCHO	CIMMYT
CWI23065[c]	ABYSSINIAN 26	ETHIOPIA
CWI23440	ELS6404.131.3	ETHIOPIA
CWI23446	ELS6404.139.7	ETHIOPIA
CWI23473	ELS6404.61.2	ETHIOPIA
CWI23385	ELS6404.84.3	ETHIOPIA
CWI21256	FELLOWS P8741	ETHIOPIA
CWI21737	HARLAN J.R 1939	ETHIOPIA
CWI22064	IAR.W.1.1	ETHIOPIA
CWI22089	IAR.W.12.2	ETHIOPIA
CWI22294	IAR.W.185.1	ETHIOPIA
CWI22102	IAR.W.22.3	ETHIOPIA
CWI22143	IAR.W.44.2	ETHIOPIA
CWI22166	IAR.W.56.2	ETHIOPIA
CWI22175	IAR.W.63.1	ETHIOPIA
CWI22201	IAR.W.84.2{	ETHIOPIA

CWI22214	IAR.W.92.1	ETHIOPIA
CWI20872	ST483	ETHIOPIA

Table S3. Intrid and cross or cultivar and seed source origin of seedling susceptible in the greenhouse, but adult plant resistant.

**Intrid[a]**	**Cross or cultivar**	**Country Origin (seed)**
G5202	T.DIC 1460/2*MEX75	CIMMYT
DW5199	IM//CIT71/CII	ETHIOPIA
CWI20826	ABISPADO DE LEBRIJA	SPAIN
DW4477	ASTURIE L2	SPAIN
CWI13539	AUSTRALIAN	INDIA
CWI21033	CANOCO	PORTUGAL
CWI19754	DICKSON J.G 417	ITALY
CWI19758	DICKSON J.G 429	PORTUGAL
CWI26558	F10 DURUM	BRAZIL
CWI22230	IAR.W.98.4	ETHIOPIA
CWI22921	I57581	USSR
CWI22927	PI57587	USSR
CWI22466	GIZA 39	UAR
CWI23095	PI61874	MOROCCO
CWI23602	QUALSET BYDV 2	USA
CWI75601	VIOLET DURUM	ETHIOPIA
DW4192	ARRANCADA-P3-13695	PORTUGAL
G7358	TDIC1458/MEX75//KHP*2/D31708	CIMMYT
G7492	FTA/CJ71//CMH76A.169/2*MEX75	CIMMYT
CWI21086	ADDIS ALEM 171	ETHIOPIA
CWI20285	BARRIGON PUBESCENT	MEXICO
CWI23075	BELADI 33	UAR
CWI22396	BENOR	FRANCE
CWI20657	HANSOTI	INDIA
CWI22951	PI57659	UAR

Intrid = CIMMYT Germplasm Bank accession number.

Table S4. Intrid and cross or cultivar and seed source of selected seedling susceptible, but adult plant resistant winter or facultative tetraploid accessions.

**Intrid[a]**	**Cross or cultivar**	**Country Origin (seed)**
DW5442	BR180/3/60.120/LDS//64.207/4/HAM	TURKEY
CWI22788	BLANCA DE NOLAS	SPAIN
DW4212	CAMBRIDGE 1	UK
CWI334	ENANO DE FACU	MEXICO
CWI22799	ENANO DE JAEN	MEXICO
CWI356	ENANO-DE-ANDUJAR	MEXICO
CWI20224	HARLAN J.R 3342	TURKEY
CWI22966	VAVILOV NI 6931	RUSSIA
CWI22789	CANA MACIZA	SPAIN
CWI22035	DF68.71	RUMANIA
CWI23196	PI68240	USSR
CWI22844	AMARELO	PORTUGAL
DW4219	CAMBRIDGE 19	UK
CWI20330	BELADI BOUHI	EGYPT

Intrid = CIMMYT Germplasm Bank accession number
